# Supplementary material for: Prevalence and determinants of healthcare avoidance during the COVID-19 pandemic: A population-based cross-sectional study
Source: PLoS Med. 2021 Nov 23;18(11):e1003854. doi: 10.1371/journal.pmed.1003854 (PMC8610236; doi:10.1371/journal.pmed.1003854)
Supplement: S1 File — (DOCX) [file pmed.1003854.s002.docx]

PROSPECTIVE ANALYSIS PLAN

**Prevalence and determinants of healthcare avoidance during the COVID-19 pandemic: a population-based, cross-sectional study**

Marije J. Splinter, Premysl Velek, M. Kamran Ikram, Brenda C.T. Kieboom, Robin P. Peeters, Patrick J.E. Bindels, M. Arfan Ikram, Frank J. Wolters, Maarten J.G. Leening, Evelien I.T. de Schepper, Silvan Licher

Date: October 2021

Version: 2.0

**Background and rationale**

During the COVID-19 pandemic, European healthcare systems were mainly focused on providing acute medical care for patients with COVID-19 (related symptoms). Most scheduled and preventive care in both the general practice and hospitals was cancelled or postponed. As a consequence, the number of consultations and diagnoses in primary care related to chronic diseases such as cancer, cardiovascular diseases, and mental illnesses, and referrals to hospitals for these indications, declined during the first six months of 2020 compared to 2019. To date, these changes in healthcare utilisation have exclusively been based on registry data of diagnoses, which only concerns patients who have actually sought medical attention for their symptoms. Detailed data from the patient’s perspective is currently absent but would provide complementary insights in the healthcare utilisation of the general population during the COVID-19 pandemic. In this period of time, individuals might have refrained from seeking medical care due to the fear of becoming infected with COVID-19 or to prevent burdening the pressured healthcare system.

In the short and medium term, the declines in consultation rates can result in severe health damage. Even though not all symptoms need direct medical evaluation, urgent assessment is necessary for some of them, such as symptoms signalling underlying cardiovascular diseases. Therefore, it is not only crucial to determine the prevalence of healthcare avoidance, but also for what symptoms healthcare was avoided and which determinants are predisposed to this behaviour, in order to mitigate health damage on the long term. The aim of this study is to expand knowledge on healthcare avoidance among the general population using data from the population-based Rotterdam Study, which could be used to develop policy interventions aimed at motivating groups of individuals most prone to avoid healthcare to timely seek medical attention for their symptoms.

Existing data on healthcare avoidance during the COVID-19 pandemic is originated from the United States, who have a general practice system that is different than the gatekeeper system that is applied in most European countries. These studies have theorised about potential determinants of healthcare avoidance, which are age, ethnic background, mental and physical comorbidities, educational level, and work status. The type of symptoms that individuals experience would also affect their decision whether or not to reach out to their physician.

**Objectives**

1. To determine the prevalence of healthcare avoidance among the general population during the COVID-19 pandemic.
2. To determine for which symptoms healthcare was avoided.
3. To assess determinants that predispose to healthcare avoiding behaviour.

**Study design**

This cross-sectional study will be embedded within the ongoing population-based Rotterdam Study, a prospective cohort study aimed at investigating the aetiology and natural history of chronic diseases in mid- and late-life. In 1990, all residents of the suburb Ommoord in the city of Rotterdam who were 55 years and older were invited to join the study. Subsequently, the cohort has been expanded multiple times: in 2000 (RS-II, lowest age limit 55), 2006 (RS-III, lowest age limit 45) and 2016 (RS-IV, lowest age limit 40) respectively. Recruited over these four study waves, the Rotterdam Study comprises 18924 participants aged 40 years and older. Among all living and non-institutionalised participants, we will send out a COVID-19 dedicated questionnaire, addressing healthcare utilisation, socioeconomic factors, mental and physical health, medication use, and COVID-19-specific symptoms.

**Study population**

The study population will consist of all participants from the Rotterdam Study who were alive on April 8^th^ 2020 and who were not hospitalised or living in a nursing home (N=8732) given the fact that these participants would be under direct and daily medical supervision of a geriatrician or nursing home physician, limiting potential healthcare avoidance.

**Primary outcome**

Prevalence of healthcare avoidance.

**Statistical analyses**

- **Baseline characteristics**: descriptive summaries of participants’ basic characteristics, stratified by healthcare avoidance (yes/no).
- **Statistical tests**: level of statistical significance will be set at 5% with corresponding 95% confidence intervals. Associations between determinants and healthcare avoidance will be calculated using binary logistic regression analysis. Data will be handled and analysed using the Statistical Package for the Social Sciences software (SPSS), version 25.0.

**Objective A:** To determine the prevalence of healthcare avoidance among the general population during the COVID-19 pandemic from a patient’s perspective.

We will assess the prevalence of healthcare avoidance by inquiring about participants’ healthcare utilisation in the previously described dedicated COVID-19 questionnaire. Moreover, we will request and analyse free-text GP records, containing narrative data of these healthcare avoiding participants to evaluate their healthcare utilisation. Since the GP is generally the first to contact when an individual experiences symptoms, healthcare avoidance will be reflected by the absence of physical, telephone, and administrative consultations in the medical records kept by the GP.

**Objective B:** To determine for which symptoms healthcare was avoided.

We will include a pre-specified list of symptoms in the COVID-19 questionnaire, so that participants can indicate for which symptoms they avoided healthcare. In the GP records, we will subsequently verify whether participants have actually refrained from seeking medical attention for these symptoms, or whether they delayed their visit.

**Objective C:** To assess determinants that predispose to healthcare avoiding behaviour.

Based on literature about healthcare utilisation, we will include several potential determinants of healthcare avoidance in the questionnaire: age, sex, occupational status, concern about contracting COVID-19, self-appreciated health, consumption of alcohol, smoking status, and the level of depression and anxiety. The association between educational level and healthcare avoidance will also be investigated; this determinant has been retrieved from earlier measurements in the Rotterdam Study from 2015 (cohorts I, II & III) and 2020 (cohort IV).
