# Supplementary material for: Prevalence and determinants of healthcare avoidance during the COVID-19 pandemic: A population-based cross-sectional study
Source: PLoS Med. 2021 Nov 23;18(11):e1003854. doi: 10.1371/journal.pmed.1003854 (PMC8610236; doi:10.1371/journal.pmed.1003854)
Supplement: S1 Tables — Table A. Characteristics of excluded participants. Table B. Characteristics of responders versus nonresponders. Table C. Determinants of healthcare avoidance stratified by potentially alarming and generic symptoms. Table D. Determinants of healthcare avoidance among participants with or without a history of any chronic disease. Table E. Determinants of healthcare avoidance stratified by different levels of healthcare avoidance. Table F. Determinants of healthcare avoidance stratified by chronic disease. (DOCX) [file pmed.1003854.s003.docx]

**S1 Tables.** Supporting tables.

| S1 Table A: Characteristics of excluded participants | 2 |
| --- | --- |
| S1 Table B: Characteristics of responders versus non-responders | 3 |
| S1 Table C: Determinants of healthcare avoidance stratified by potentially alarming and generic symptoms | 4 |
| S1 Table D: Determinants of healthcare avoidance among participants with or without a history of any chronic disease  S1 Table E: Determinants of healthcare avoidance stratified by different levels of healthcare avoidance | 5  6 |
| S1 Table F: Determinants of healthcare avoidance stratified by chronic disease | 7 |

| **S1 Table A \| Characteristics of participants with incomplete data on healthcare utilisation (N=585). Values are numbers (percentages) unless stated otherwise** | | |
| --- | --- | --- |
| **Determinants** | |  |
| Age, years (mean, SD) | | 72.7 (12.7) |
| Women | | 379 (64.8) |
| History of chronic diseases | Any | 156 (26.7) |
|  | Cancer | 40 (6.8) |
|  | Heart disease | 62 (10.6) |
|  | Stroke | 27 (4.6) |
|  | Chronic lung disease | 32 (5.5) |
|  | Neurodegenerative disease | 2 (0.3) |
|  | Diabetes | 24 (4.1) |
|  | Mental illness | 10 (1.7) |
| Educational level | Primary education | 66 (11.3) |
|  | Low/intermediate general or lower vocational | 220 (37.8) |
|  | Intermediate vocational or higher general | 170 (29.2) |
|  | Higher vocational or university | 119 (20.4) |
| Self-appreciated health | Poor | 7 (1.2) |
|  | Fair | 30 (5.1) |
|  | Good | 144 (24.6) |
|  | Very good | 41 (7.0) |
|  | Excellent | 16 (2.7) |
| Occupation | Working (full time, part time, self-employed) | 40 (6.8) |
|  | On sick leave | 2 (0.3) |
|  | Unemployed | 8 (1.4) |
|  | Retired | 162 (27.7) |
|  | Other | 9 (1.5) |
| Alcohol consumption; yes | | 107 (18.3) |
| Current smoking; yes | | 15 (2.6) |
| Concern contracting COVID-19 | Never | 36 (6.0) |
|  | Rarely | 59 (9.9) |
|  | Sometimes | 116 (19.5) |
|  | Often | 21 (3.5) |
|  | Almost continuously | 2 (0.3) |
| Symptoms of depression (weighted score ≥ 10) | | 54 (9.2) |
| Symptoms of anxiety (weighted score ≥ 7) | | 47 (8.0) |
| Abbreviations: N = number of participants, SD = standard deviation  1.23% of all values were missing. | | |

| **S1 Table B \| Characteristics of non-responders (N=2491) versus responders (N=6241). Values are numbers (percentages) unless stated otherwise** | | | |
| --- | --- | --- | --- |
| **Determinants** | | **Non-responders** | **Responders** |
| Age, years (mean, SD) | | 68.9 (14.0) | 70.2 (11.6) |
| Women | | 1519 (61.0) | 3643 (58.0) |
| Educational level | Primary education | 304 (12.2) | 343 (6.1) |
|  | Low/intermediate general or lower vocational | 914 (36.7) | 1875 (33.2) |
|  | Intermediate vocational or higher general | 741 (29.7) | 1807 (31.9) |
|  | Higher vocational or university | 513 (20.6) | 1579 (27.9) |
| Native Dutch ethnic background |  | 2085 (87.0) | 5709 (94.0) |
| Abbreviations: N = number of participants, SD = standard deviation | | |  |

| **S1 Table C \| Determinants of healthcare avoidance stratified by potentially alarming and generic symptoms (N=5656)** | | | |
| --- | --- | --- | --- |
|  | | **Model 1** | **Model 2** |
|  | | **Odds ratio (95% CI)** | **Odds ratio (95% CI)** |
| Age, per 10 years increase | | 1.28 (1.17-1.40)** | 1.19 (1.11-1.27)** |
| Women | | 1.74 (1.40-2.16)** | 1.51 (1.28-1.78)** |
| Educational level vs. higher vocational or university | Primary education | 1.98 (1.28-3.05)** | 1.78 (1.27-2.50)** |
|  | Low/intermediate general or lower vocational | 1.40 (1.05-1.88)* | 1.18 (0.94-1.47) |
|  | Intermediate vocational or higher general | 1.17 (0.88-1.57) | 1.27 (1.03-1.57)* |
| Self-appreciated health, per level decrease | | 3.05 (2.59-3.60)** | 1.80 (1.61-2.02)** |
| Occupational status vs. employed | Retired | 1.57 (1.05-2.35)* | 1.12 (0.84(1.49) |
|  | Unemployed | 2.89 (1.59-5.28)** | 2.17 (1.38-3.43)** |
| Alcohol consumption; yes | | 0.64 (0.52-0.79)** | 0.87 (0.74-1.02) |
| Current smoking; yes | | 1.26 (0.90-1.75) | 1.39 (1.09-1.78)** |
| Concern contracting COVID-19, per level increase | | 1.55 (1.37-1.74)** | 1.23 (1.13-1.35)** |
| Weighted level of depression, per score increase | | 1.17 (1.15-1.20)** | 1.11 (1.09-1.12)** |
| Weighted level of anxiety, per score increase | | 1.22 (1.19-1.25)** | 1.13 (1.11-1.16)** |
| Abbreviations: CI = confidence interval, N = number of participants.  *p < 0.05; **p < 0.01  All models are adjusted for age and sex.  Model 1: binary logistic regression analyses among participants who reported one or more potentially urgent symptoms: chest pain, limb weakness, palpitations, difficulty speaking or facial drooping, and self-perceived cancer-related symptoms.  Model 2: binary logistic regression analyses among participants who only reported symptoms of a more generic nature: lower back pain, sudden onset dizziness, memory complaints, fluid retention (oedema), elevated blood pressure, attempts to stop or reduce smoking, nausea and/or vomiting, sudden (temporary) vision loss, and dysregulation of diabetes. | | | |

| **S1 Table D \| Determinants of healthcare avoidance among participants with or without a history of any chronic disease (N=5561)** | | | |
| --- | --- | --- | --- |
| **Determinants** | | **Odds ratio (95% CI)**  **Without any chronic disease (N=1900)** | **Odds ratio (95% CI)**  **With any chronic disease (N=3661)** |
| Age, per 10 years increase | | 1.03 (0.92-1.16) | 1.22 (1.15-1.29)** |
| Women | | 1.42 (1.08-1.88)* | 1.59 (1.38-1.82)** |
| Educational level vs. higher vocational or university | Primary education | 1.49 (0.78-2.84) | 1.85 (1.39-2.46)** |
|  | Low/intermediate general or lower vocational | 1.08 (0.74-1.58) | 1.26 (1.04-1.51)* |
|  | Intermediate vocational or higher general | 1.33 (0.95-1.86) | 1.23 (1.03-1.48)* |
| Self-appreciated health, per level decrease | | 1.86 (1.52-2.28)** | 2.13 (1.93-2.35)** |
| Occupational status vs. employed | Retired | 1.37 (0.86-2.18) | 1.26 (0.99-1.61) |
|  | Unemployed | 1.75 (0.88-3.51) | 2.37 (1.60-3.51)** |
| Alcohol consumption; yes | | 0.87 (0.66-1.15) | 0.78 (0.68-0.89)** |
| Current smoking; yes | | 1.31 (0.87-1.96) | 1.35 (1.09-1.66)** |
| Concern contracting COVID-19, per level increase | | 1.15 (0.98-1.34) | 1.33 (1.24-1.43)** |
| Weighted level of depression, per score increase | | 1.13 (1.10-1.16)** | 1.13 (1.12-1.15)** |
| Weighted level of anxiety, per score increase | | 1.17 (1.12-1.21)** | 1.17 (1.14-1.19)** |
| Abbreviations: CI = confidence interval, N = number of participants.  *p < 0.05; **p < 0.01  All models are adjusted for age and sex. | | | |

| **S1 Table E \| Determinants of healthcare avoidance stratified by different levels of healthcare avoidance (N=889)** | | | |
| --- | --- | --- | --- |
| **Determinants** | | **Odds ratio (95% CI)**  **Definite and probable (N=668)** | **Odds ratio (95% CI)** |
|  |  |  | **Possible (N=221)** |
| Age, per 10 years increase | | 1.24 (1.16-1.34)** | 1.14 (1.03-1.27)* |
| Women | | 1.72 (1.44-2.05)** | 1.57 (1.20-2.04)** |
| Educational level vs. higher vocational or university | Primary education | 2.08 (1.43-3.02)** | 1.13 (0.66-1.93) |
|  | Low/intermediate general or lower vocational | 1.40 (1.09-1.80)** | 1.04 (0.74-1.47) |
|  | Intermediate vocational or higher general | 1.52 (1.20-1.93)** | 0.80 (0.57-1.13) |
| Self-appreciated health, per level decrease | | 2.27 (1.99-2.59)** | 2.05 (1.71-2.46)** |
| Occupational status vs. employed | Retired | 1.06 (0.77-1.47) | 1.39 (0.87-2.23) |
|  | Unemployed | 2.71 (1.68-4.37)** | 1.01 (0.39-2.66) |
| Alcohol consumption; yes | | 0.78 (0.66-0.93)** | 0.77 (0.60-0.99)* |
| Current smoking; yes | | 1.32 (1.01-1.72)* | 1.19 (0.79-1.78) |
| Concern contracting COVID-19, per level increase | | 1.30 (1.18-1.43)** | 1.36 (1.18-1.58)** |
| Weighted level of depression, per score increase | | 1.14 (1.12-1.15)** | 1.11 (1.08-1.13)** |
| Weighted level of anxiety, per score increase | | 1.17 (1.14-1.20)** | 1.13 (1.09-1.17)** |
| Abbreviations: CI = confidence interval, N = number of participants.  *p < 0.05; **p < 0.01  All models are adjusted for age and sex. | | | |

| **S1 Table F \| Determinants of healthcare avoidance stratified by chronic disease.** | | | | | | |  |  |
| --- | --- | --- | --- | --- | --- | --- | --- | --- |
| **Determinants** | | **Odds ratio (95% CI)**  **Heart disease (N=1640)** | **Odds ratio (95% CI)**  **Cancer (N=812)** | **Odds ratio (95% CI)**  **Chronic lung disease (N=795)** | **Odds ratio (95% CI)**  **Diabetes (N=547)** | **Odds ratio (95% CI)**  **Stroke (N=418)** | **Odds ratio (95% CI)**  **Mental illness (N=257)** | **Odds ratio (95% CI)**  **Neurodegenerative disease (N=97)** |
| Age, per 10 years increase | | 1.19 (1.07-1.33)** | 1.23 (1.05-1.45)* | 1.16 (1.01-1.33)* | 1.23 (1.02-1.49)* | 1.30 (1.06-1.61)* | 0.88 (.070-1.11) | 1.01 (0.68-1.51) |
| Women | | 1.77 (1.41-2.23)** | 1.33 (0.96-1.84) | 1.45 (1.05-2.01)* | 1.90 (1.30-2.78)** | 2.02 (1.31-3.10)** | 0.88 (0.51-1.49) | 0.83 (0.35-1.98) |
| Educational level vs. higher vocational or university | Primary education | 2.16 (1.34-3.48)** | 1.37 (0.62-3.05) | 2.07 (1.14-3.77)* | 1.81 (0.77-4.26) | 1.52 (0.62-3.73) | 2.50 (1.68-3.74)** | 2.29 (0.39-13.5) |
|  | Low/intermediate general or lower vocational | 1.16 (0.84-1.59) | 1.64 (1.03-2.62)* | 1.57 (0.99-2.48) | 0.99 (0.56-1.76) | 0.97 (0.51-1.82) | 0.95 (0.46-1.96) | 1.01 (0.23-4.56) |
|  | Intermediate vocational or higher general | 0.98 (0.71-1.35) | 1.47 (0.93-2.32) | 1.08 (0.68-1.72) | 0.84 (0.48-1.49) | 0.73 (0.38-1.38) | 0.84 (0.42-1.69) | 1.33 (0.41-4.35) |
| Self-appreciated health, per level decrease | | 2.01 (1.74-2.43)** | 1.82 (1.45-2.29)** | 2.10 (1.65-2.68)** | 1.71 (1.30-2.26)** | 1.84 (1.35-2.50)** | 2.50 (1.68-3.74)** | 1.28 (0.70-2.33) |
| Occupational status vs. employed | Retired | 1.21 (0.78-1.88) | 0.78 (0.41-1.49) | 1.51 (0.84-2.73) | 2.66 (1.17-6.01)* | 2.99 (0.88-10.10) | 2.46 (0.84-7.25) | n.a. |
|  | Unemployed | 3.53 (1.59-7.81)** | 0.83 (0.21-3.36) | 3.53 (1.45-8.62)** | 6.60 (1.73-25.09)** | n.a. | 2.84 (0.97-8.28) | n.a. |
| Alcohol consumption; yes | | 0.73 (0.58-0.92)** | 0.84 (0.61-1.16) | 0.68 (0.49-0.93)* | 1.02 (0.68-1.52) | 0.88 (0.57-1.36) | 0.67 (0.39-1.12) | 0.78 (0.31-1.95) |
| Current smoking; yes | | 1.42 (0.96-2.10) | 1.16 (0.66-2.07) | 1.48 (0.95-2.31) | 1.57 (0.84-2.93) | 1.99 (1.00-3.97)* | 1.42 (0.76-2.65) | 1.16 (0.27-5.00) |
| Concern contracting COVID-19, per level increase | | 1.32 (1.16-1.51)** | 1.24 (1.05-1.47)* | 1.38 (1.16-1.64)** | 1.32 (1.08-1.61)** | 1.47 (1.14-1.88)** | 1.52 (1.17-2.02)** | 0.85 (0.53-1.36) |
| Weighted level of depression, per score increase | | 1.14 (1.12-1.17)** | 1.11 (1.08-1.15)** | 1.12 (1.08-1.15)** | 1.17 (1.12-1.23)** | 1.12 (1.07-1.17)** | 1.11 (1.06-1.16)** | 1.02 (0.94-1.11) |
| Weighted level of anxiety, per score increase | | 1.18 (1.14-1.22)** | 1.14 (1.09-1.20)** | 1.14 (1.09-1.19)** | 1.16 (1.10-1.23)** | 1.18 (1.11-1.26)** | 1.12 (1.06-1.18)** | 0.98 (0.87-1.11) |
| Abbreviations: CI = confidence interval, N = number of participants, n.a. = not applicable (number of cases lower than 5).  *p < 0.05; **p < 0.01  All models are adjusted for age and sex. | | | | | | |  |  |
