# Supplementary material for: Prevalence and determinants of healthcare avoidance during the COVID-19 pandemic: A population-based cross-sectional study
Source: PLoS Med. 2021 Nov 23;18(11):e1003854. doi: 10.1371/journal.pmed.1003854 (PMC8610236; doi:10.1371/journal.pmed.1003854)
Supplement: Attachment — Submitted filename: Review Comment to manuscript PMEDICINE-D-21-02940R1 rev.HS.-converted.pdf [file pmed.1003854.s004.pdf]

Review Comment to manuscript PMEDICINE-D-21-02940R1 - [EMID:154a3e6dbe90f9d1]

Prevalence and determinants of healthcare avoidance during the COVID-19 pandemic: a population-based study

Title is good and complete. There was dependent variable and independent variable that reflect the material topic and the objective of the study

Abstract. Good complete.. suitable with the core of the study. It could be minimize but it is still informative.

Methods

This is not prospective cohort study but it is cross sectional survey, that was apart of the ongoing population-based Rotterdam Study, a prospective cohort study.

using primary and secondary data, it is ok, but which one is going to be used? or is combine data going to be used as an eligibility? criteria of the study.?

It needs clearly eligibility of the study for sampling such as age, not hospitalised or living in nursing homes, etc, it’s has been written

more accurate in methods as a sample criteria of the study.

Was questionare valideted? should be explained.

Respons rate is better to write in the result of the study not in the methods

It is better to be shorted

Result, discussion, and conclusion was excellent…. added suggestions in conclusion is better. so that immediate action is taken regarding the benefits

Heath care . it shoud be highlight to prevent the damage for vulnerale group because of avoiding health care. do not wait to the damage happens.......It has been shown in implication better for **suggestion**

Review Summarize

This is an excellent article, an original article that deserves to be published and would be very useful both in the short and long term.

It’s clinically applicative and heath community important
We often hear, the questions in this study, does the patient's fear of COVID-19 cause the avoidance of seeking help? of course, this avoidance can also pose a danger to the patients.

It is also possible that these conditions may also present in health care providers.

The researcher realizes with excellent research, tries to reduce the bias that may occur. Presented in a completely and systematically and also conducted excellent and adequate analysis.
This research is easy to read and to understand, it would be very useful for many interests, in many countries that are also experiencing a pandemic COVID-19

Reviewer. H. Shatri MD
